# Guillain–Barré syndrome in a 63-year-old patient possibly triggered by ehrlichiosis. Case report

**DOI:** 10.1016/j.ijregi.2024.100422

**Published:** 2024-08-13

**Authors:** Carlos Mejía-Irías, Jaqueline Hernández-Posadas, Meyling Zapata, Nelson Mercadal

**Affiliations:** Centro Médico Olanchano (CMO), Olancho, Juticalpa, Honduras

**Keywords:** Guillain–Barré syndrome, Acute autoimmune neuropathy, Ehrlichiosis

## Abstract

Guillain–Barré syndrome is an immune-mediated acute demyelinating polyradiculoneuropathy, characterized by progressive flaccid weakness, triggered mainly by respiratory and gastrointestinal infections. We present the case of a 63-year-old male patient with a history of *Ehrlichia* infection, who consulted the internal medicine emergency department for lower back pain and progressive lower limb paresthesia, accompanied by decreased lower limb strength and nerve conduction velocity test, with results compatible with acute demyelinating sensorimotor polyradiculoneuropathy. To the best of our knowledge, this is the first documented case in Honduran medical literature; in our research, no other cases were found in Latin America or Europe. The importance of the topic and its dissemination in countries where *Ehrlichia* infection exists is that when cases of Guillain–Barré syndrome that cannot be associated with previous gastrointestinal or respiratory infection, they could be attributed to *Ehrlichia* infection as a possible cause; therefore, exhaustive preventive measures can be established regarding the transmitting vector of ehrlichiosis.

## Highlights

• *Ehrlichia* infection as a possible trigger for Guillain–Barré syndrome.
• First-line treatment failure (doxycycline) in the case report patient.
• Therapeutic success for *Ehrlichia* infection using rifampicin in case report patient.

## Introduction

Guillain–Barré syndrome is an acute immune-mediated demyelinating polyradiculoneuropathy that induces rapid and progressive flaccid weakness [[1], [2], [3], [4], [5]]. Triggered by an aberrant autoimmune response to a previous infection in two-thirds of cases or other immune stimulation, causing the immune system to attack myelin sheaths due to molecular mimicry [3,4,6,7]. The first cases were described by Landry in 1859 and by Guillain, Barré, and Stroh in 1916 [2,7,8].

Guillain–Barré syndrome occurs worldwide, with an incidence of one to two cases per 100,000 people per year. It affects all age groups [3,[5], [6], [7], [8]], with a slightly higher incidence in men [3,4,[9], [10], [11]]. In Honduras, according to Sánchez et al. [11] at Hospital Escuela Universitario, 30 (32.9%) cases were reported in 2016; 22 (24.2%) cases in 2017; 33 (36.3%) in 2018; and six (6.6%) until February 2019.

Respiratory or gastrointestinal tract infections are the most common triggers of Guillain–Barré syndrome, preceding it in 75% of cases [3,5,12]. It has also been associated with cases of ehrlichiosis, such as one reported in a 71-year-old woman in Arkansas and a 66-year-old man with coinfection with *Babesia microti*, Epstein–Barr virus, and *Arcobacter butzleri* [1,5,12].

We present the first case documented in Honduran literature of Guillain–Barré syndrome associated with *Ehrlichia* infection. The importance of the issue in countries where *Ehrlichia* infection exists is that when cases of Guillain–Barré syndrome occur that cannot be associated with gastrointestinal or respiratory infection, they could be attributed to *Ehrlichia* infection as a possible trigger; in this way, exhaustive preventive measures regarding the transmitting vector of ehrlichiosis can be established.

## Case report

We present the case of a 63-year-old male patient from Juticalpa, Olancho, who works as a farmer and rancher and has pets at home. The patient is prediabetic, with a history of tick bite in inguinal region 1 month before consultation. The patient presented with a headache lasting 3 days, polyarthralgia, chills, and dysesthesia in lower limbs. Simple brain tomography with normal findings for his age. In addition, a peripheral blood smear examination with Wright stain was requested and sent to a laboratory in Tegucigalpa, with a processing time of 7 days. Upon arriving home after the consultation, he tripped and received trauma on the sacral region, without seeking medical attention for 7 days. Then, he consulted the emergency department for intense low back pain, accompanied by progressive reduction of lower limb strength and exacerbation of paresthesia.

He was received with pain in the paravertebral region at L3-S1 level, with paretic gait, preserved tendon reflexes, and grade IV strength in the lower limbs according to Daniels and Worthingham's muscle grading scale. In addition, a peripheral blood smear was received, finding intracellular morulae, positive for *Ehrlichia*. He was admitted to the hospital with analgesics and doxycycline 100 mg every 12 hours.

During the 3^rd^ day of admission, he was evaluated by the neurosurgery service for the described symptoms, onset of paresthesia, and distal-to-proximal dysesthesia in the upper limbs, for which a cervical and lumbosacral magnetic resonance imaging was requested; the findings included C5-C6 cervical spondylosis with normal spinal cord and L3-L4 and L4-L5 central rachystenosis, with no indication for surgical treatment.

On the 5^th^ day of hospitalization, he presented a distal-to-proximal grade III decrease in strength on his lower limbs and absent tendon reflexes on four limbs; a lumbar puncture and nerve conduction velocity test were performed. Cytochemistry of cerebrospinal fluid reported four cells, total proteins 32.9 mg/dL (Table 1). Nerve conduction velocity test was compatible with acute demyelinating sensory motor polyradiculoneuropathy of all four extremities.

**Table 1:** Laboratory test results.

Variable	Result	Reference range (adults)
Hemoglobin (g/dL)	14.7	12-16.5
White blood cells	10,100	4500-10,500
Platelets	201,000	150,000-450,000
Total creatine phosphokinase test (U/L)	112.5	39.0-308.0
Aspartate aminotransferase (U/L)	13.9	0.0-40.0
Alanine aminotransferase (U/L)	23.7	0.0-40.0
Potassium (mEq/L)	4.9	3.5-5.3
Calcium (mg/dL)	9.48	8.8-11.0
Creatinine (mg/dL)	0.98	0.70-1.20
Blood urea nitrogen (mg/dL)	14.55	6.0-20.0
VIH1/VIH2 antibodies	Negative	
Blood smear	Intracytoplasmic morula positive *Ehrlichia*	
Glycated hemoglobin	6.3%	5.7-6.4 %
Thyroid-stimulating hormone (µUI/mL)	2.25	0.27-4.20
Cerebrospinal fluid		
Cytochemistry		
Cells (células/uL)	4	<5
Glucose (mg/dL)	95.39	45-80
Proteins (mg/dL)	32.9	15-45
Cerebrospinal fluid glucose/serum glucose ratio	0.68	
Gram	No bacteria present	
Bacteria culture	Negative	
India ink stain	Negative for mycotic structure	
Ziehl-Neelsen	Negative	
Gen Xpert	Negative, *Mycobacterium tuberculosis* not present	
Adenosine deaminase	7 U/L	<9 U/l

He was prescribed human immunoglobulin 38 g IV every day for 5 days, and gabapentin 300 mg every 8 hours was started. During the second day of treatment, he had episodes of sympathetic dysautonomia, for which he required morphine on three occasions. At the end of treatment with human immunoglobulin, he had an improvement in strength on his lower limbs, with grade IV on the Daniels and Worthingham's muscle grading scale and reported absence of pain and paresthesia in her upper and lower limbs. He was discharged 4 days after finishing treatment with human immunoglobulin, completing 14 days of treatment with doxycycline 100 mg every 12 hours and began physical therapy.

The patient was re-evaluated in the outpatient clinic 2 months later, managing to walk with a cane and without paresthesia. In the third month after discharge, a peripheral blood smear control was requested, in which *Ehrlichia* persisted; therefore, treatment was started with rifampicin 300 mg every 12 hours for 10 days. Subsequently, 6 months after discharge, the patient was able to walk independently, without sequelae; a peripheral blood smear examination was repeated, in which the presence of *Ehrlichia* was no longer found.

## Discussion

Guillain–Barré syndrome is characterized by progressive, distal-to-proximal symmetrical muscle weakness, accompanied by hyporeflexia or areflexia [1,3,4,9,11], which can be determined through diagnostic criteria established in Brighton, published in 2011 [9,13,14]. We sensory signs observed in our patient, such as lumbar region pain, accompanied by progressive, symmetrical bilateral decrease in strength; cerebrospinal fluid studies showed no albuminocytological dissociation and nerve conduction velocity that reported findings compatible with acute demyelinating sensory motor polyradiculoneuropathy. Clinical correlation with laboratory data is crucial; in this case, the patient corresponds to 50% of patients who do not develop albuminocytological dissociation after the first week of symptom onset.

The most common triggers of Guillain–Barré syndrome are infectious diseases of the respiratory or gastrointestinal tract [2,9,14], triggering a T-cell–mediated immune response. For instance, in *Campylobacter jejuni* infection, the lipooligosaccharide in the outer membrane of the bacteria bears a resemblance to gangliosides of peripheral nerves, causing a cross-reaction that leads to the attack of peripheral nerves and myelin sheaths [1].

However, our research on PubMed found two reports of ehrlichiosis associated with the development of Guillain–Barré syndrome [1,15]. Our case would be the third case to present this association because the presence of *Ehrlichia* infection was established through the detection of intracellular morulae, and, subsequently, Guillain–Barré Syndrome was diagnosed (Table 2). We found no studies focused on the pathophysiologic mechanisms, specifically, during *Ehrlichia* infection. There are studies that mention canine postinfectious polyradiculoneuritis after *Ehrlichia* infection, which could be suggested as a similar pathogenesis for Guillain–Barré syndrome in humans [16]. We recommend further studies into the specific pathophysiologic mechanisms for the development of Guillain–Barré syndrome associated with *Ehrlichia* infection in humans.

**Table 2:** Comparative table: case studies associating Guillain–Barré syndrome and *Ehrlichia*.

Case report	Case report: Malhis et al. [1]	Case report: Farrington et al. [15]	Current case report
Patient age (years)	71	66	63
Area	South central USA	North-eastern USA	Honduras, Central America
*Ehrlichia* diagnostic test used	Polymerase chain reaction	Immunoglobulin M/G	Peripheral blood smear wright
Coinfection	No	Yes, *Babesia microti*, Epstein–Barr, *Arcobacter butzler*	No
Antibiotic treatment	Doxycycline 100 mg BID (12 days)	Doxycycline 100 mg BID (10 days); azithromycin 500 mg BID (10 days); atovaquone 750 mg BID (10 days)	Doxycycline 100 mg BID (14 days); then rifampicin 300 mg BID (10 days)
Days from *Ehrlichia* onset to GBS development	3 weeks	3 weeks	2 weeks
Cerebrospinal fluid albuminocytological dissociation	Yes	No	No
Intensive care unit admission	No	Yes	No
Ambulation recovery	Not mentioned	3 months follow-up with a cane	2 months follow-up with a cane; independent 6 months follow-up

The first-line treatment for ehrlichiosis is doxycycline for 14 days; no resistance has been reported in the literature. In our case, the patient received doxycycline for 14 days. A total of 2 months after finishing treatment, intracellular morulae persisted. thus, it was necessary to start treatment with rifampicin for 10 days. After repeating the peripheral blood smear, no more intracytoplasmic morulae were found. Subsequent control tests to confirm the eradication of the infection and identify possible cases of antibiotic therapy resistance should be emphasized.

Regarding patient recovery, the literature states that 85% of patients regain independent ambulation with early treatment initiation [1,2]. In our case, the patient achieved full recovery after 6 months, being able to ambulate independently and return to his daily and work activities.

In conclusion, Guillain–Barré syndrome can be associated with previous respiratory or gastrointestinal infectious diseases; however, in our case, it was linked to *Ehrlichia* infection. Therefore, in regions where tick exposure exists and no other causes for the development of Guillain–Barré syndrome can be identified, ehrlichiosis should be considered as a potential etiologic factor and treatment should be provided once identified.

## Declarations of competing interest

The authors have no competing interests to declare.
